# Tackling Cultural Determinants of Health Through Nutrition Education Among Refugees

**DOI:** 10.1089/heq.2020.0109

**Published:** 2021-06-01

**Authors:** Abiodun T. Atoloye, Habiba Nur, Heidi Wengreen, Martha Archuleta

**Affiliations:** ^1^ Rudd Center for Food Policy and Obesity, University of Connecticut, Hartford, Connecticut, USA.; ^2^ Department of Nutrition, Dietetics, and Food Sciences, Utah State University, Logan, Utah, USA.; ^3^ Department of Nutrition, Dietetics, and Food Sciences, Salt Lake Center, Utah State University, Logan, Utah, USA.

**Keywords:** refugees, nutrition education, culture, cultural determinants

## Abstract

Nutrition education provides an avenue to address nutrition behavior change needs and prevents chronic disease in refugee communities. Previous studies have consistently identified cultural uniqueness as one of the barriers to meeting the needs of the refugee communities effectively. This current perspective describes the barriers and opportunities to improve nutrition education efforts among this population while taking into account the cultural context.

## Introduction

Refugee communities are disproportionately affected by food insecurity and chronic diseases compared with the general population of the host country.^1,2^ Refugee communities are uniquely characterized by diversity in language, cultural attitude and belief, education, and literacy level.^2^ These characteristics in addition to limited access to resources such as adequate income, information, and transportation may amplify the barriers that refugees experience in their attempt to access healthy food.^3,4^ Furthermore, refugees are exposed to factors that make it difficult to navigate their new food environment and make healthy food choices.^3,4^ These factors include increased exposure to unhealthy food options through food advertisements, low-cost foods with low nutritional value, and poor access to familiar foods.^4^ For example, some refugees who had prior healthier eating behavior may feel the need to acculturate to the abundant and enticing calorie-dense food in their new environment. These calorie-dense foods are often easy to obtain and lower cost than more nutrient-dense or familiar options. Put together, these factors contribute to poor nutrition and health disparity, including a high risk for chronic diseases.^5^

Nutrition education has been used as a strategy for preventing obesity and other diet-related chronic diseases such as diabetes.^6,7^ Among the general population, nutrition education plays a significant role through behavioral change, by increasing self-efficacy and motivating healthier food-related decisions.^7,8^ Similarly, nutrition education can improve dietary practices and nutrition outcomes among the refugee population.^9^ However, despite the wide recognition that culture can strongly influence eating behavior, nutrition, and health outcomes among refugees, nutrition education efforts targeting the refugee communities have not always included a culturally sensitive approach or model.^10,11^ Recognition of the cultural context in nutrition education can improve its effectiveness in reducing nutrition and health inequities in refugee communities.

We recently conducted a nationwide survey among nutrition education practitioners in the United States^10^ and a review of the literature on current practices in the delivery of nutrition education among refugees.^12^ In this study, we summarize and discuss the key themes from the survey and review challenges and opportunities for the delivery of culturally appropriate and sensitive nutrition education that may inform strategic efforts to advance health equity among refugee communities.

## Challenges in Delivering Culturally Sensitive Nutrition Education Programs to Refugees

### Lack of standardized and validated nutrition education strategies and tools

A wide range of implementation and evaluation tools have been used in nutrition education programs that target refugees. For example, oral matching quizzes,^13^ food purchase receipts,^14^ interviews,^15^ Automated Self-Administered 24-hour Recall (ASA24),^16^ direct observations, and weighing^17^ have been used to assess program outcomes, but none of these have been validated for use among refugees. Similarly, a variety of curricula and instructional delivery strategies have been used to address cultural sensitivity in nutrition education delivery. These include translated resources,^18^ translations by refugee children or community members,^19,20^ pictures,^21,22^ simple words,^14,18^ and hands-on cooking classes.^17^ Some of these strategies are promising in addressing the language barrier, delivering appropriate literacy-level lessons, and reducing the response burden among the target audience. However, none of the aforementioned strategies or tools have been validated for use among refugees. This lack of standardized/validated strategies and tools precludes proof of their effectiveness.

### Lack of cultural competency training to address the varied refugee diet and lifestyle

Despite widespread recognition that cultural competency—a behavior and attitude that ensure that a system or individual effectively and appropriately functions in diverse cultural interaction and settings^23^—can address health inequality, nutrition educators are inadequately trained in this regard. Therefore, the delivery of culturally appropriate and sensitive nutrition education to effectively meet the needs of these audiences is challenged. Our review found only a few studies that focused on providing training to educators to enhance the delivery of nutrition education among refugees.^16,24,25^ Among the three studies only one included cultural competency training among different refugee service providers such as caseworkers, employment advocates, and English as a Second Language instructors.^24^ More focus was on other competency training such as community-based participatory research partnership orientation, family-focused communication, human research participant training, social cognitive theory, motivational interviewing, nutrition-disease links, behavioral change, and intervention delivery principles.^16,24,25^ Although there is limited evidence of the impact of this training on dietary outcomes among refugees recent studies have called attention to the need for skill-based training on cultural competence among nutrition education practitioners.^10,11^

Furthermore, although translators and interpreters improve communication and interaction between practitioners and the target audience, this strategy does not guarantee messages are delivered within the cultural context of the audience.

## Recommendations

Although a standardized program evaluation tool and resources such as the ASA24 are beneficial among the general population, the subtle cultural differences among refugee groups preclude the unification of resources and evaluation tools used among refugee communities. A more flexible and audience-specific evaluation strategy could be effective. With refugee communities, nutrition education resources and program evaluation tools should align with the audience's specificity and must be responsive to contextual differences among the target groups. Hence nutrition educators should adapt nutrition education resources and their evaluation activities to meet the changing contexts among the target population. This might require a more comprehensive evaluation planning that includes collaboratively: (1) assessing the evaluation need and (2) developing the evaluation activities with the community members. For example, the target group should work collaboratively with nutrition educators to determine the types of questions to ask and how the variable of interest can be captured using content- and tone-appropriate tools.

To address the lack of coherence between the existing resources and the need for flexibility, a comprehensive best practice tool kit that provides evidence-based strategies that have worked for professionals and institutions serving refugee communities needs to be developed. For practice-based purposes, this would serve as a go-to resource that could provide valuable ideas for implementing or sustaining nutrition education programs. The resource should provide program practitioners with information to help them effectively understand refugee communities. It should also provide a menu of useful resources such as curricula, evaluation instruments, and other aids to help them address needs that may arise in their work with refugee communities. Similar tools have been developed for addressing the needs of a diverse older adult population^26^ and encouraging better care among health care providers.^27^

A common way to address the cultural capacity of professionals and organizations is through training and workshops on how to be culturally sensitive.^28^ In addition to gaining knowledge on refugee cultures, strategies for effectively engaging with refugees in a culturally appropriate way are needed as a key element in cultural competency training. Otherwise, cultural competence training could increase cultural awareness and knowledge but may fail to generate defined cultural responsiveness needed to meet the needs of the varying refugee communities served in practice.^29^ Moreover, refugee populations need health and nutrition practitioners who can resonate with their unique experiences and needs. This requires that educators build a rapport of trust and support with the refugees that they serve. Therefore, nutrition educators need to use a relationship-based approach and relying on shared connections to develop the trust needed. This calls for a peer-educator model for offering a credible and culturally sensitive nutrition education among refugees. Refugees possess an implicit understanding of their cultural dynamics that formal training in cultural competence may not provide or produce. Therefore, in addition to cultural competence training among professionals, collaboration with the refugee community itself and their involvement as nutrition educators is imperative to better tailor efforts to meet the specific needs of the target audience.

It is worthy to note that research on refugees' nutrition education is still in its early stage and would benefit greatly from approaches that will be inclusive of refugees in the planning, implementation, and evaluation processes. In conclusion, the awareness and consideration of the cultural contexts that influence health-related beliefs, attitudes, and behaviors are needed to optimize nutrition outcomes and reduce nutrition and health inequities among refugee communities.

## Abbreviation Used

ASA24: Automated Self-Administered 24-hour Recall
